# Identification of* Raoultella terrigena* as a Rare Causative Agent of Subungual Abscess Based on 16S rRNA and Housekeeping Gene Sequencing

**DOI:** 10.1155/2016/3879635

**Published:** 2016-06-09

**Authors:** Yu Wang, Xiawei Jiang, Zemin Xu, Chaoqun Ying, Wei Yu, Yonghong Xiao

**Affiliations:** ^1^State Key Laboratory for Diagnosis and Treatment of Infectious Diseases, Collaborative Innovation Center for Diagnosis and Treatment of Infectious Diseases, The First Affiliated Hospital, School of Medicine, Zhejiang University, Hangzhou, China; ^2^Ningbo Institute of Microcirculation and Henbane, Ningbo, China

## Abstract

A 63-year-old-man was admitted to our hospital with severe subungual abscess. Bacteria were isolated from pus samples, and an inconsistent identification was shown by VITEK 2 system and MALDI-TOF mass spectrometry as* Raoultella planticola* and* Raoultella terrigena*, respectively. Molecular identification by 16S rRNA sequencing suggested that the isolate is* R. terrigena*, and this was further demonstrated by sequencing three housekeeping genes (*rpoB*,* gyrA*, and* parC*) with phylogenetic analysis. To our knowledge, this is the first report of subungual abscess caused by* R. terrigena*, a rare case of human infection due to soil bacterium. Our study highlights the technique importance on this pathogen identification.

## 1. Introduction

*Raoultella terrigena* is a Gram-negative, oxidase-negative, aerobic, nonmotile, capsulated, non-spore-forming bacterium. It was primarily recovered from soil and considered as a nonpathogenic species, although it harbours numerous virulence factors found in* Klebsiella pneumoniae* [1]. Very few reports of clinical infections caused by this species are available so far, and its clinical significance is still unclear [1]. A previous study showed that the carriage rate of* R. terrigena* in healthy people was 0.9% by analyzing 5,377 different stool specimens [2]. The incidence of* R*.* terrigena* among clinical* Klebsiella* isolates was 0.4%, and most of these isolates were obtained from respiratory secretions [2]. We here reported a case of a 63-year-old man who developed a subungual abscess caused by* R. terrigena*. We further performed a literature review on* R. terrigena* infections.

## 2. Case History

In March 2015, a 63-year-old man was admitted to the surgery department of a university hospital with the complaint of a painful abscess on his right thumb lasting for 3 days. The patient is a farmer and had no prior medical and surgical history. Eight days before his admission, the patient experienced an accidental injury on his thumb during working in the field and later washed the wound by river water. As shown by physical examination, the abscess was at the lateral nail edge, and finger pad was tense and pus presented. No other skin lesions were detected. The patient was consequently diagnosed as the subungual abscess. A longitudinal incision and drainage were thus performed with expression of purulent fluid, and a culture of the fluid was performed. The relief of pain immediately after this treatment was reported by the patient. 500 mg of imipenem twice a day for 10 days was prescribed to the patient, and he recovered after this treatment.

**Figure 1:**
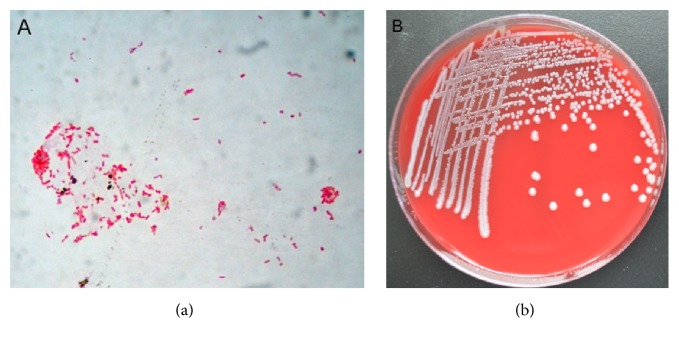
Gram staining and morphology of* R. terrigena* Z38 colony. (a) Gram-negative, short-rod-shaped bacterium was observed (magnification, ×100). (b) Shown are circular, smooth, glistening, light yellow, and nonhemolysis colonies after a 48-hour culture on sheep blood agar under aerobic conditions.

The pus from the incision was inoculated onto sheep blood agar plates and incubated at 37°C in a CO_2_-enriched (5%) atmosphere for 24 h. Incubation yielded single colonies of Gram-negative rod-shaped bacterium identified by Gram staining (Figure 1(a)). Colonies of isolate Z38 were circular, smooth, glistening, light yellow, and nonhemolysis after 48 h of incubation on sheep blood agar plates at 37°C (Figure 1(b)). The samples were analyzed by MALDI-TOF mass spectrometry (Autoflex III, Bruker, Germany) and were identified as* Raoultella terrigena* with a similarity rate of 99.9%.

As* Raoultella terrigena* is rarely identified in clinical samples, identification was subsequently redone by VITEK 2 system with panel AST-GN-13 (BioMérieux, France). The isolate was identified as* Raoultella planticola* by the VITEK 2 system. Antimicrobial susceptibility testing was also performed by VITEK 2 system with panel AST-GN-13, and* Escherichia coli* ATCC 25922 and* Pseudomonas aeruginosa* ATCC 27853 were as controls. The result interpretation was performed as described in CLSI guidelines. The isolate was susceptible to all antibiotics tested except ampicillin (Table 1).

To get a reliable identification, 16S rRNA and the housekeeping gene sequencing were further used to accurately identify and discriminate. PCR amplification of the 16S rRNA was performed with the universal bacterial primers 27f (5′-AGAGTTTGATCCTGGCTCAG-3′) and 1492r (5′-TACCTTGTTACGACTT-3′). The 1.4 kb PCR product was sequenced and blasted in GenBank to identify the species. Alignments of 16S rRNA sequences of the isolate Z38 and type strains of close related species collected from EzTaxon-e database were generated by ClustalW program. A neighbor-joining phylogenetic tree based on the Tamura-Nei model was constructed by MAGA6.06. The phylogeny indicated the strain Z38 (KT276326) sharing 99% identity with the type strain* Raoultella terrigena* NBRC 12941^T^ (Y17658) (Figure S1A in Supplementary Material available online at http://dx.doi.org/10.1155/2016/3879635).

Housekeeping genes* rpoB*,* gyrA*, and* parC* were sequenced to demonstrate the taxonomic identification as described previously [3]. The sequence similarity of* gyrA*,* parC*, and* rpoB* between pus isolate Z38 and the most closely related type strain (*R. terrigena* NBRC 12941^T^) was 99%, 99%, 98%, respectively (Table 2 and Figures S2, S3, and S4). The phylogenetic tree constructed by concatenating sequences of the 3 housekeeping genes showed that strain Z38 formed a distinct lineage with* R. terrigena* NBRC 12941^T^ (Figure S1B). Together with the typing result of 16S rRNA, the pus isolate Z38 was identified as* R. terrigena*.

## 3. Discussion

*R. terrigena* is a soil microorganism with only two reports of clinical infection. Goegele et al. described the first case of human infection in a liver transplant recipient who developed fatal endocarditis due to* R. terrigena* in 2007 [4]. The association between* R. terrigena* and sepsis was reported in 2011, which represents the second case of human infection [5]. We speculated that the pathogen in our case may originate from environment, since the patient injured in the farmland and had the risk of infection by soil bacterium.

Accurate identification of* Raoultella* spp. is a challenge by routine work as this genus shares highly similar phenotypes with other genera of Enterobacteriaceae, especially* Klebsiella* spp. [5]. In 2001,* Raoultella* spp. as a novel species was separated from* Klebsiella* spp. on the basis of 16S rDNA and* rpo*B typing results [6]. Phylogenetic analysis of* gyr*B sequences showed that* K. terrigena* (*R. terrigena*) and* K. pneumoniae* belong to two separate lineages [7]. Difficulties in correct identification of* Raoultella* spp. may have led to an underestimation of its incidence and uncertainty on its pathogenic role [8]. Currently, the identification of* Raoultella* species from clinical specimens mainly relied on phenotypic methods. A study has validated that MALDI-TOF MS increases the accuracy of* Raoultella* identifications to the genus level [9]. However, the three species of* Raoultella* genus shared very close genetic relationship and can only be distinguished by a very few biochemical characters. In our study, a discrepancy was caused by MALDI-TOF mass spectrometry and VITEK 2 identification. Thus, molecular techniques are highly recommended to discriminate the three species [5].

However, no molecular markers are available for this purpose from the literature due to the rarity of the pathogen. A study showed that* rpoB* was demonstrated on the correct identifications of all* Raoultella* isolates [8]. Furthermore, three housekeeping genes* gyrA* (383 bp),* rpoB* (512 bp), and* parC* (319 bp) were used for species-level identification of members of the* Klebsiella/Raoultella* complex [3]. Therefore, in our study three housekeeping genes (*gyrA*,* rpoB*, and* parC*) were chosen for the identification of* R. terrigena*. The phylogenetic analysis by concatenating the 3 housekeeping genes showed that strain Z38 was clustered with* R. terrigena*. This result is consistent with the results of MALDI-TOF and 16S rRNA sequencing (Figure S1). We therefore concluded that VITEK 2 system is not suitable for* R. terrigena* identification. Our study further implies that human infections by* R. terrigena* may be underestimated due to the difficulties in correctly identifying this bacterium during routine diagnosis in clinical microbiology laboratories.

The clinical features and outcomes of human infections caused by* R. terrigena* have been rarely reported. The first* R. terrigena* infection case was reported in 2007, and a 45-year-old patient developed endocarditis due to* R. terrigena* after liver transplant [4]. Another infection case occurred in a patient who underwent pancreatic resection for pancreatic cancer [5]. However, the pathogenic role of* Raoultella* spp. in human infection is still difficult to elucidate. Notably, some species of* Raoultella *spp., for example,* Raoultella ornithinolytica*, have been suggested as opportunistic pathogens that mainly infect elderly patients with immunosuppression or comorbidities, particularly solid tumours [8].

In conclusion, this report describes the first case of subungual abscess caused by* R. terrigena* infection. Our finding highlights that the soil microflora enables causing severe infections. Additionally, molecular identification enables us to accurately identify fastidious organisms from clinical specimens when phenotypic identification fails.

## Supplementary Material

The neighbor-joining trees of the pus isolate Z38 to other close related species and the neighbor-joining trees of Z38 based on the comparative analysis of rpoB, gyrA, and parC sequences.

## Figures and Tables

**Table 1:** Antibiotic resistance profiles of *Raoultella terrigena* Z38.

Antibiotics	MIC (*μ*g/mL)^a^	Susceptibility
Ampicillin	16	R
Amikacin	≤2	S
Ciprofloxacin	≤0.25	S
Levofloxacin	≤0.25	S
Cefoperazone	≤1	S
Imipenem	≤1	S
Trimethoprim-sulfamethoxazole	≤20	S
Tobramycin	≤1	S
Piperacillin-tazobactam	≤4	S
Ampicillin-sulbactam	≤2	S
Aztreonam	≤1	S
Cefotetan	≤4	S
Cefazolin	≤4	S
Gentamicin	≤1	S
Ceftazidime	≤1	S
Cefepime	≤1	S
Ertapenem	≤0.5	S

^a^MICs were determined using the Vitek 2 GN and AST cards, following the manufacturer's instructions. The susceptibility breakpoints of MICs followed those recommend by the Clinical and Laboratory Standards Institute.

**Table 2:** The list of gene sequences used for phylogenetic tree construction.

Species	16S rRNA	*gyrA*	*rpoB*	*parC*
*Raoultella terrigena* NBRC 12941^T^	Y17658	AF303617	AY367362	AF303651
*Raoultella planticola* NBRC 12939^T^	AF129443	AF303621	AY367361	AF303655
*Raoultella ornithinolytica* NBRC 105727^T^	AF129441	AF303618	AF129447	AF303652
*Raoultella electrica* 1GB^T^	AB762091	AB828204	AB828205	AB828206
*Klebsiella oxytoca* ATCC 13182^T^	U78183	AF052257	AY367363	—
*Klebsiella oxytoca* KCTC 1686	NR_102982	CP003218	CP003218	CP003218
*Klebsiella michiganensis* W14^T^	JQ070300	JQ990329	JQ269337	—
*Klebsiella variicola* At-22	NR_074729	NC_013850	NC_013850	CP001891
*Klebsiella pneumoniae* subsp. *pneumonia* ATCC 13883^T^	Y17656	DQ673325	DQ673324	AF303641
*Klebsiella pneumoniae* subsp. *rhinoscleromatis* ATCC 13884^T^	NR_114507	FO203501	ACZD01000183	ACZD01000120
*Klebsiella pneumoniae* subsp. *ozaenae* ATCC 11297	AF130982	AF303619	AF129445	AF303653
*Serratia liquefaciens* ATCC 27592^T^	NR_121703	CP006252	NC_021742	CP006252
