# Supplementary material for: Identification of Raoultella terrigena as a Rare Causative Agent of Subungual Abscess Based on 16S rRNA and Housekeeping Gene Sequencing
Source: Can J Infect Dis Med Microbiol. 2016 Jun 9;2016:3879635. doi: 10.1155/2016/3879635 (PMC4917704; doi:10.1155/2016/3879635)

Supplemental Materials

Figure S1. Neighbor-joining tree showing the phylogenetic position of the pus isolate Z38 to other close related species. A. NJ tree was constructed based on the 16s rRNA genes. B. NJ tree was constructed based on concatenated sequences (*gyrA+parC+rpoB*, 1214 bp). Bootstrap consensus trees were inferred from 100 replicates, only bootstrap values > 50% were indicated. The sequences of *Serratia liquefaciens* ATCC 27592^T were used as outgroups. GenBank accession numbers are given in parentheses. Bar, 0.5% and 2% nucleotide substitution rate.

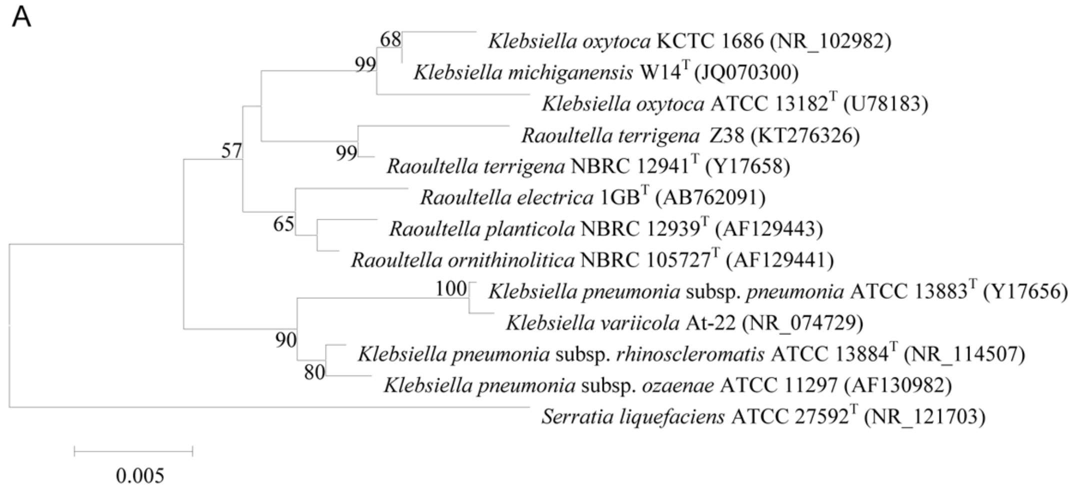

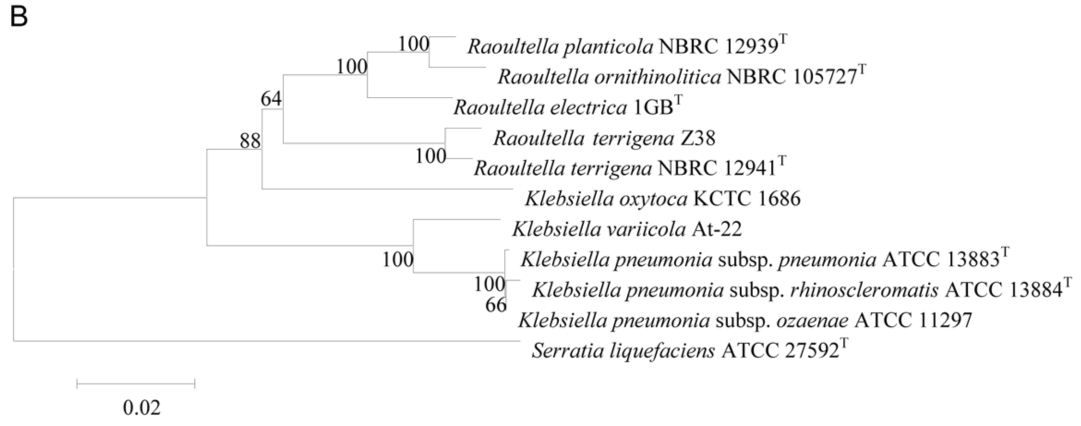

Figure S2. Neighbor-joining tree showing the phylogenetic position of the pus isolate Z38 based on the comparative analysis of *gyrA* sequences. All position containing alignment gaps and missing data were eliminated only in pairwise sequence comparisons.

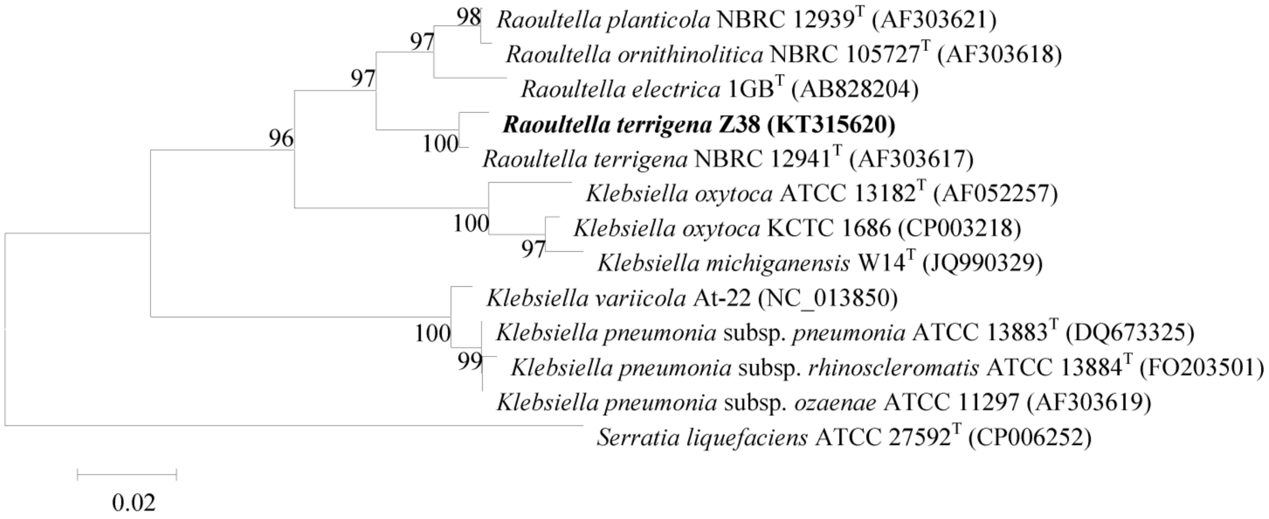

Figure S3. Phylogenetic trees constructed from comparative analysis of *parC* sequences showing the relationship of the pus isolate Z38 with other related species. These trees were made using the neighbor-joining method. All position containing alignment gaps and missing data were eliminated only in pairwise sequence comparisons.

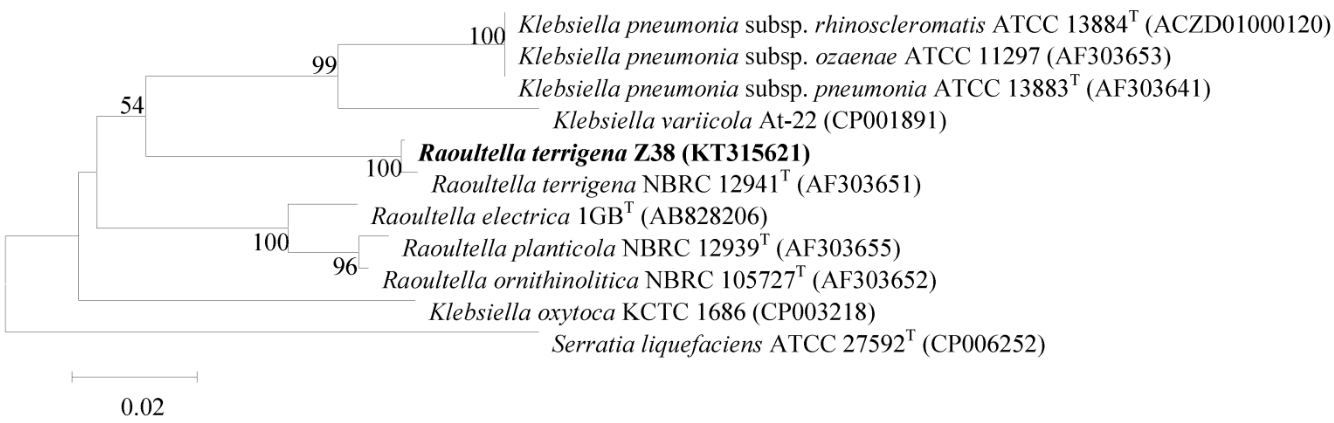

Figure S4. Phylogenetic trees constructed from comparative analysis of *rpoB* sequences showing the relationship of the pus isolate Z38 with other related species. These trees were made using the neighbor-joining method. All position containing alignment gaps and missing data were eliminated only in pairwise sequence comparisons.

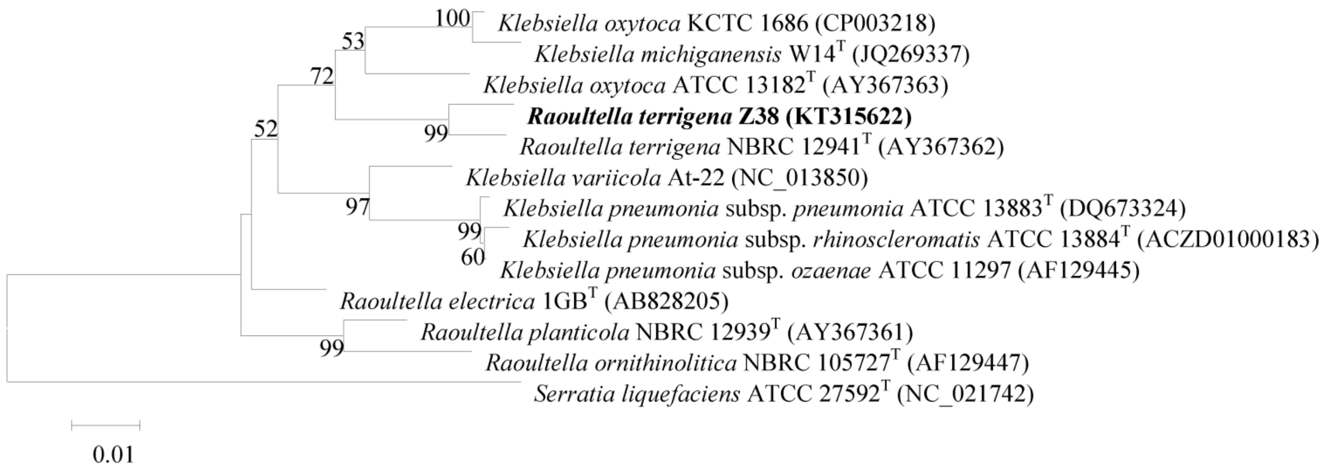

Supplement: Supplementary file 1 — The neighbor-joining trees of the pus isolate Z38 to other close related species and the neighbor-joining trees of Z38 based on the comparative analysis of rpoB, gyrA, and parC sequences. [file 3879635.f1.pdf]
